# Complete genomes of *Rickettsia typhi* reveal a clonal population

**DOI:** 10.1371/journal.pntd.0013828

**Published:** 2025-12-29

**Authors:** Chantisa Keeratipusana, Weerawat Phuklia, Vanheuang Phommadeechack, Janjira Thaipadungpanit, Vilada Chansamouth, Koukeo Phommasone, Sayaphet Rattanavong, Catrin E. Moore, Matthew T. Robinson, Allen L. Richards, Paul N. Newton, Elizabeth M. Batty

**Affiliations:** 1 Mahidol-Oxford Tropical Medicine Research Unit, Faculty of Tropical Medicine, Mahidol University, Phaya Thai, Bangkok, Thailand; 2 Lao-Oxford-Mahosot Hospital-Wellcome Trust Research Unit, Microbiology Laboratory, Mahosot Hospital, Vientiane, Lao PDR; 3 Department of Clinical Tropical Medicine, Faculty of Tropical Medicine, Mahidol University, Bangkok, Thailand; 4 Centre for Neonatal and Paediatric Infection, Institute for Infection and Immunity, City St. George’s, University of London, London, United Kingdom; 5 Centre for Tropical Medicine and Global Health, Nuffield Department of Medicine, University of Oxford, Oxford, United Kingdom; 6 Naval Medical Research Center, Infectious Disease Directorate, Forest Glen, Maryland, United States of America; 7 Uniformed Services University of the Health Sciences, Preventive Medicine and Biostatistics Department, Bethesda, Maryland, United States of America; Yale University School of Medicine, UNITED STATES OF AMERICA

## Abstract

Murine typhus, caused by infection with *Rickettsia typhi*, is a neglected disease contributing to infectious disease burden in south- and southeast Asia. Despite its importance, we have minimal knowledge of the genomics of *R. typhi*, with only four complete genomes being sequenced prior to this work. We sequenced a further 25 genomes including historical strains collected before 1976 from both human and rat hosts, and recent genomes isolated from patients at a single hospital in Laos. Whole genome SNP analysis reveals extremely low levels of genetic diversity across the 29 genomes, with overall nucleotide diversity (π) of 1.27e^-05^ and evidence of purifying selection, and a minimal pan-genome. Phylogenetic analysis shows clustering of the genome by historic or modern origin, with the exception of one modern strain which is most closely related to historic strains from Thailand, and no clustering by host origin. The highly conserved genome of *R. typhi* suggests strong constraints on genome evolution in this obligate intracellular parasite, and has implications for the design of future murine typhus diagnostic tools and vaccines.

## Author summary

*Rickettsia typhi* is the bacteria which causes murine typhus, an infectious disease transmitted by fleas. It is often underdiagnosed because its symptoms are similar to many other illnesses. To better understand this pathogen, we examined a large collection of *R. typhi* genomes taken from recent patient infections and historical bacterial collections covering almost a century. The findings showed that *R. typhi* has evolved very slowly over time. Even across nations, hosts, and decades, its genetic composition is extremely stable. This implies that the population of the bacteria is highly uniform and their genetics differ very little from one another. This low level of genetic variation will help in the design of effective *R. typhi* diagnostic tests, and simplify future vaccine development.

## Introduction

*Rickettsia typhi* causes murine typhus, a neglected infectious disease contributing to the burden of febrile illness and associated morbidity and mortality in humans, especially in south- and southeast Asia [1]. It is transmitted by the bites of infected fleas, which typically live on rats [2]*.* Murine typhus occurs worldwide and while mortality is low at approximately 0.4-3.6% [3], morbidity in untreated disease can be significant, showing the importance of early diagnosis and treatment.

All species of the genus *Rickettsia* are obligate intracellular bacteria associated with arthropod hosts, and can be classified into four major groups: 1) the typhus group, which includes *R. typhi* and *Rickettsia prowazekii*, that cause murine typhus and epidemic typhus, with flea and louse hosts, respectively; 2) the spotted fever group, which includes *Rickettsia rickettsii, Rickettsia japonica* and *Rickettsia conorii,* found in ticks; 3) the ancestral group of *Rickettsia bellii* and *Rickettsia canadensis,* which are non-virulent species found in ticks; and 4) the transitional group rickettsiae which include: *Rickettsia akari, Rickettsia australis* and *Rickettsia felis*, which are mite-, tick- and flea-borne, respectively [4].

The first complete genome of *R. typhi* was sequenced in 2004, revealing 877 genes in a 1.11Mb genome [5]. Most *Rickettsia* species contain a single rRNA operon composed of the 16S, 23S, and 5S rRNA genes. This configuration is consistent with their relatively low replication rates and adaptability to a stable intracellular environment. *Rickettsia* species share a single rRNA operon, with little variation [6]. Further comparative genomics of complete rickettsial genomes defined five phylogenetic groups, with the typhus group having the smallest genome size of the five groups. While plasmids were identified in many rickettsial species, no plasmids have been identified in any of the typhus group strains, although *R. prowazekii* has a gene cluster thought to result from integration of an ancestral *Rickettsia* plasmid into the chromosome [7]. *R. typhi* is naturally sensitive to rifampin, but other *Rickettsia* species in the spotted fever group show resistance, and a limited number of studies have investigated laboratory-derived rifampin resistance in *R. typhi* [8–10].

While the first genome was published over twenty years ago, our knowledge of *R. typhi* genomics remains minimal, likely due to low rates of diagnosis and the difficulty in culturing and sequencing bacteria from patients even when a confirmed diagnosis is available [11]. Previous work has compared four complete genomes and identified a small number of genetic changes between strains collected over a long timeframe [12]. The mutations detected in three of these genomes were used as probes to type a further 15 isolates of *R. typhi*, and to generate a phylogeny based on 26 SNPs and indels [13].

In this study we used whole genome sequencing to expand our knowledge on the genomes of *R. typhi* from two populations: a set of eleven historic strains collected from seven countries between 1928 and 1975, from the collection of the Naval Medical Research Center, USA, and a set of seventeen samples collected over ten years (2006–2019) from Mahosot Hospital in the Lao PDR (Laos) [11]. Using these datasets we were able to compare historic data to modern strains, and describe the diversity found in patient isolates collected over a ten-year timeframe in one country.

## Materials and methods

### Ethical statement

Ethical approvals for collection of samples for determination of the etiology of fever in Lao were granted by the Lao National Ethics Committee for Health Research and the Oxford Tropical Research Ethics Committee. Sequencing was carried out from strains previously isolated from patient specimens during routine microbiological investigations. Strains were grown in commercial cell lines and no sequencing was performed from direct patient samples, therefore ethical approval for sequencing of isolates was not required as no patient sample or patient DNA was present.

### *Rickettsia typhi* culture and DNA extraction

For the samples cultured from patients with murine typhus at Mahosot Hospital, up to 5 ml of blood was drawn into EDTA tubes after obtaining written informed consent from patients. Full isolation procedure is detailed in Ming *et. al.* [11]. Briefly, either 1 mL whole EDTA anticoagulated blood, or 200 μL EDTA buffy coat fraction was mixed with 5 mL of RPMI 1640 medium supplemented with 10% foetal calf serum (FCS) (PAA Laboratories, USA, or Gibco, USA). Inoculation was performed directly in 12.5 cm^2^ flasks containing L929 (ATCC number CCL-1) cell monolayers at 80% confluence, and subsequently centrifuged for 30 minutes at 50 × *g*. Flasks were incubated for 2 hours at 35°C in a 5% CO_2_ atmosphere and then the inoculum washed and replaced with RPMI 1640 with 10% FCS. Flasks were incubated for a minimum of 8 weeks at 35°C in a 5% CO_2_ atmosphere, with a twice-weekly media change and sub-culture performed at week four. Cells were checked for successful isolation by indirect IFA using pooled antibodies against scrub typhus, spotted fever and typhus group rickettsial agents every week after 4 weeks of incubation or earlier [14]. Cultures positive by IFA underwent DNA extraction using DNeasy Blood & Tissue Kit (Qiagen, UK) and isolation was confirmed by qPCR targeting the *R. typhi* 17kDa gene [15].

For genomic DNA (gDNA) extraction, infected cells from three 75-cm^2^ flasks were mechanically detached, resuspended and transferred to a 50-mL conical-bottom tube and centrifuged at 3,220 x g for 10 minutes. The pellet was resuspended in 3-mL fresh medium and transferred to 1.5 mL microcentrifuged tubes. Tubes were vortexed for one minute and host cell debris was removed after centrifugation at 300 x g for 3 minutes. The supernatant was passed through a 2.0 μM filter (Paradisc, GE Whatman, USA). Subsequently, 10 μL of 1.4 μg/μL DNAse was added per 1 mL of bacteria solution, incubated at room temperature for 30 minutes. The mixture was centrifuged at 18,188 x g for 10 minutes, the pellet was washed twice with 0.3M sucrose (Sigma-Aldrich). DNA was extracted from the *R. typhi* pellet using the DNeasy Blood & Tissue kit (Qiagen, Hilden, Germany) following the manufacturer’s protocol. DNA was eluted in AE buffer and stored immediately at -20°C. The yield of genomic DNA was quantified using a nanodrop spectrophotometer (Nanodrop 2000, Thermo Scientific, UK). The ratio between *R. typhi* and host cells was determined using qPCR of the *R. typhi ompB* [16] and mouse *GADPH* genes.

### Illumina sequencing

Sequencing libraries were prepared from extracted DNA using the Illumina Nextera Flex library preparation kit (Illumina, USA) according to manufacturer’s protocols. Libraries were barcoded using the Illumina DNA Tagmentation indexes (Illumina, USA) and pooled and sequenced on a single run of a MiSeq instrument using the MiSeq V3 paired-end 300 bp sequencing kit.

### Bioinformatic analysis

Three public assembly genomes and 25 Illumina short reads were analysed using the Bactopia v2.2.0 pipeline [17]. Variants were called and annotated using freeBayes v1.3.6 [18] and snpEff v5.2 [19], respectively. Consensus data with only substitution variants were used to create a phylogenetic tree with 1000 bootstrap replicates using Iqtree v2.2.5 [20]. The tree was plotted, annotated and visualized using ggtree v3.10.0 [21]. BactDating [22] was used to assess the evolutionary rate, and SNPgenie [23] to estimate nucleotide diversity and selection pressure. Illumina short reads were *de novo* assembled using Skesa [24]. After *de novo* assembly, contigs showing similarity to the mouse genome with a 99% identity threshold match using BLAST v2.15.0 [25] were removed. BUSCO v5.7.1 [26] using the OrthoDB version10 bacterial dataset was used to assess the gene content. Prokka v1.14.6 [27] was used to annotate both the existing *R. typhi* genomes and the new assemblies. Panaroo v1.3.4 [28] was used to construct a pan-genome with strict stringency mode and core-genome sample threshold of 1. TM2540 assembly data was improved by TM2540 Illumina sequencing data in this study using Pilon v1.24 [29]. The polished TM2540 assembly data was compared to the original TM2540 by the dnadiff option from MUMmer v3.23 [30].

## Results

We generated whole genome sequencing data from two populations of *R. typhi*: a set of eleven historic strains collected from seven countries between 1928 and 1975, and a set of fourteen strains collected over ten years (2006–2019) from a single hospital in Laos (Table 1). This second set included resequencing of the previously sequenced TM2540 isolate [12]. We included three complete genomes that were previously made available in our analysis (TH1527, B9991CWPP, and Wilmington/NC_006142.1), and the Wilmington strain was used as the reference genome. The date and country of origin for each strain is shown in Table 1. Whole genome sequencing statistics are shown in Table 2. Coverage depth ranged from 37X to 294X. 1%-11% of reads were determined to be mouse contamination, which was expected as the *R. typhi* strains were cultured in L929 mouse fibroblast cells.

**Table 1:** Isolate information table.

Isolates	Host	Country	Year	Reference
NC_006142.1/Wilmington	Human	USA	1928	Maxcy, 1928 [31]
GEAR	Human	South Africa	1939	Gear, 1954 [32]
GER	Human	Azerbaijan	1946	Eremeeva et al, 1996 [33]
MUSSEIBOV	Human	Azerbaijan	1949	Eremeeva et al, 1996 [33]
FLA6590	Rat	USA	1951	Perez Gallardo and Fox, 1948 [34]
TA837	Rat	Thailand	1963	WRAIR, Bozeman/Sangkasuwan
TH1527	Human	Thailand	1965	WRAIR, Bozeman/Sangkasuwan
PAKNA	Rat	Pakistan	1970	University of Maryland, Dasch/Wisseman
B9991CWPP	Rat	Myanmar	1970	
AZ306	Rat	Ethiopia	1975	University of Maryland, A. Azad, personal communication
AZ331	Rat	Ethiopia	1975	University of Maryland, A. Azad, personal communication
TM1041	Human	Vientiane Capital, Laos	2006	
TM1377	Human	Vientiane Capital, Laos	2007	
TM2418	Human	Vientiane Capital, Laos	2008	
TM2522	Human	Vientiane Capital, Laos	2009	
TM2607	Human	Vientiane Capital, Laos	2009	
TM2529	Human	Vientiane Capital, Laos	2009	
TM2557	Human	Vientiane Capital, Laos	2009	
TM2540	Human	Bolikhamxay Province, Laos	2009	
TM2509	Human	Vientiane Capital, Laos	2009	
TM3627	Human	Vientiane Capital, Laos	2010	
TM4034	Human	Vientiane Capital, Laos	2011	
TM3905	Human	Vientiane Capital, Laos	2011	
TM4237	Human	Vientiane Capital, Laos	2012	
TM4105	Human	Khammouan Province, Laos	2012	
TM4234	Human	Vientiane Capital, Laos	2012	
TM8956	Human	Vientiane Capital, Laos	2017	
TM10184	Human	Vientiane Capital, Laos	2019	

**Table 2:** Paired-end sequencing data summary.

SampleID	Total reads	Total bp	Average coverage	Mouse contamination (%)
GEAR	697356	50209632	44.53	4.48
GER	643912	146811936	130.22	1.44
MUSSEIBOV	498752	76309056	67.68	7.19
FLA6590	731361	220139661	195.26	2.19
TA837	567534	170827734	151.52	5.69
PAKNA	507024	122192784	108.38	2.27
AZ306	496021	82339486	73.03	11.28
AZ331	383915	61042485	54.14	5.68
TM1041	418013	46399443	41.15	6.07
TM1377	291070	87321000	77.45	2.75
TM2418	600367	55834131	49.52	2.97
TM2522	1043787	130473375	115.73	9.52
TM2607	576194	129067456	114.48	1.58
TM2529	541959	163129659	144.69	9.62
TM2557	4282437	201274539	178.53	4
TM2540	484054	100683232	89.3	1.22
TM2509	274033	64123722	56.87	7.69
TM3627	569573	116192892	103.06	5.22
TM4034	629420	116442700	103.28	9.38
TM3905	2549838	331478940	294.02	7.23
TM4237	553326	42606102	37.79	7.46
TM4105	630702	78207048	69.37	2.65
TM4234	504777	126194250	111.93	2.5
TM8956	781969	162649552	144.27	5.22
TM10184	861641	204208917	181.13	6.67

### Variant calling and phylogeny

**Fig 1:**
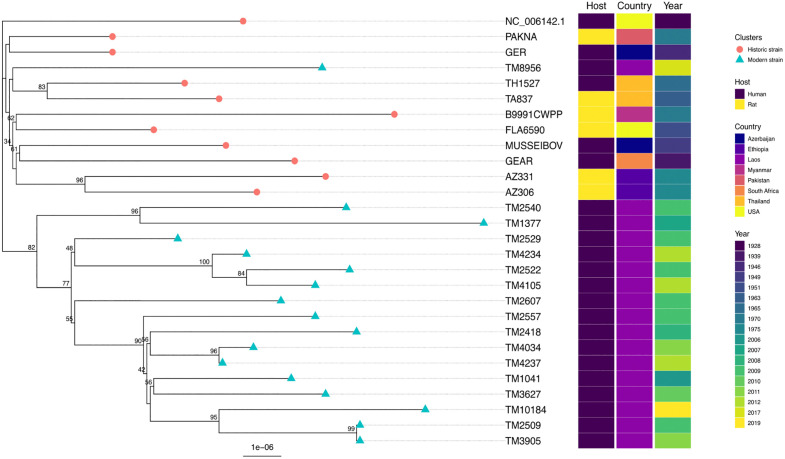
Phylogenetic tree of 28 *Rickettsia typhi* isolates. The phylogenetic tree was constructed using Iqtree with 1,000 bootstrap replicates. Figure indicates bootstrap support. The scale bar shows the substitution rate. The tree was plotted, annotated, and visualized by ggtree.

We identified 153 single nucleotide polymorphisms (SNPs) among the 28 strains of *R. typhi*. The majority of variants were annotated as missense variants compared to the Wilmington reference strain (Table 3). A phylogenetic tree generated from the 153 SNPs found in 28 *R. typhi* strains (Fig 1), shows that the isolates cluster into two distinct groups: the historic group from 1928 to 1975, and the modern group from 2006 to 2019. One isolate of the modern group, TM8956, was placed in the historic group instead of the modern group, and clusters most closely with two isolates from Thailand. The two isolates from Thailand (TH1527 and TA837) and Ethiopia (AZ330 and AZ306) form geographical clusters, while the other historic strains with the same geographic origin do not cluster together. Strains also do not cluster by their original host, with the rat samples separated across the tree, emphasizing the cross-species nature of this intracellular organism.

**Table 3:** Variant annotation of 27 *R. typhi* samples.

Annotation	N	Percentage (%)
Intergenic region	38	24.84
Missense variant	78	50.98
Stop retained variant	1	0.65
Synonymous variant	36	23.53
Total	153	100

Kato *et. al*. [13] genotyped SNPs and indels identified using the three complete genomes in a wider set of strains, which includes the AZ306, AZ331, TA837, PAKNA (also called NA18), MUSSEIBOV, GER, GEAR, and FLA6590 strains which were also sequenced in our study. We compared our variant calls across 22 SNPs and two small indels (i3_162 and i10_825) identified by Kato *et al*. [13], and the calls were identical across the strains shared between the two studies. With the extra SNPs we identify in this study it is possible to distinguish the PAKNA and GER strains, which cannot be separated using the smaller Kato set of SNPs and indels.

**Fig 2:**
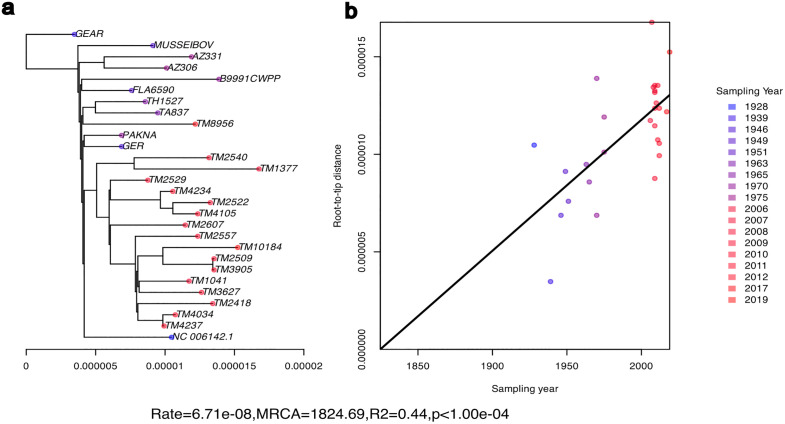
Date-scaled phylogenetic analysis of *Rickettsia typhi.* **a)** Phylogenetic tree generated using Iqtree showing substitution rate on the x-axis. Tree tips are coloured according to sampling date for each strain, with the blue to red gradient showing earliest to most recent strains. **b)** Root-to-tip regression showing a weak association between sampling date and root-to-tip distance.

We looked for clock signals in the mutation rate in *R. typhi* using BactDating which dates the nodes of a bacterial phylogenetic tree*.* Fig 2 shows the root-to-tip distance generated from the phylogenetic tree. Although there is a weak correlation between sampling date and root-to-tip distance, the signal is not significant after testing against a model with equal clock dates. Model comparison using Deviance Information Criterion (DIC) values showed a lower value for the clock model (DIC = 0.00) than the no-clock (equal-dates) model (DIC = 0.37), indicating that the clock model does not substantially improve the fit over the null model, suggesting insufficient evidence for a consistent molecular clock signal in this dataset.

Nucleotide diversity and selection pressures were estimated by SNPgenie. The results show that the overall nucleotide diversity (π) is low at 1.27e^-05^ overall, indicating limited genetic variation within the population. The nonsynonymous diversity (πN) of 1.14e^-05^ is slightly lower than synonymous diversity (πS) of 1.28e^-05^, suggesting purifying (negative) selection.

We identified one isolate, GEAR, with a nucleotide substitution in the *rpoB* gene, which encodes the RNA polymerase beta subunit and has previously been implicated in rifampin resistance. We observered an nonsynoymous A to C substitution at genomic position 164,354 resulting with the replacement of asparagine with histidine at residue 1039. This mutation does not correspond to the amino acid substitutions previously observed in the rifampin-resistant *rpoB* gene mutants generated in the laboratory [8]. However, the transition from asparagine to histidine introduces a larger side chain residue into *rpoB.*

### Assembly and genome annotation

The Illumina short-read FASTQ files were *de novo* assembled using Skesa and annotated using Prokka. Contigs which showed 99% or greater similarity to the mouse genome using BLAST were assumed to be culture contaminants and were removed. The assembly statistics for each isolate are shown in Table 4.

**Table 4:** Assembly and annotation statistics.

Isolate	Contigs	Size (bp)	Non-mouse contigs	Size(bp) after mouse contamination removal	N50	CDS	tRNA	rRNA units
TM4237	11	1109632	9	1108517	274951	861	33	3
TM4105	10	1110196	8	1108685	273807	863	33	3
TM4034	7	1108600	7	1108600	274951	863	33	3
TM4234	6	1110100	6	1110100	656226	865	33	3
TM2522	18	1118980	5	1109506	656775	864	33	3
TM2607	6	1109456	6	1109456	274951	862	33	3
TM2529	7	1126548	5	1109394	314979	864	33	3
TM3627	14	1111656	10	1108893	248264	861	33	3
TM3905	28	1135971	5	1109340	656202	863	33	3
TM2557	14	1115564	7	1109043	274951	862	33	3
TM8956	11	1111254	7	1108880	620152	861	33	3
TM2540	9	1109880	8	1109298	160875	863	33	3
TM10184	21	1128700	8	1109070	274951	862	33	3
TM1041	14	1112308	10	1109445	187840	863	33	3
AZ306	15	1130205	6	1109970	389013	863	33	3
AZ331	9	1125476	7	1109262	274951	863	33	3
FLA6590	10	1123985	6	1109047	574761	863	33	3
GEAR	14	1115426	9	1109679	274951	864	33	3
GER	16	1121520	11	1109339	220491	864	33	3
PAKNA	9	1126848	7	1109757	248253	862	33	3
MUSSEIBOV	9	1110795	7	1108930	274951	861	33	3
TA837	10	1111092	9	1110293	275097	865	33	3
TM2418	7	1111286	5	1109330	620201	864	33	3
TM1377	11	1110450	10	1109441	275504	865	33	3
TM2509	10	1110369	8	1109130	274918	862	33	3
NC_006142.1	1	1111496	1	1111496	1111496	865	33	3
TH1527	1	1112372	1	1112372	1112372	864	33	3
B9991CWPP	1	1112957	1	1112957	1112957	864	33	3

The gene content was assessed using BUSCO (S1 Fig). The number of complete genes in the assemblies is the same as for the Wilmington, TH1527, and B9991CWPP reference: 86.3%, 0.8%, and 12.9% for complete, fragmented, and missing genes, respectively, indicating that our assemblies are likely complete in gene content in comparison to these complete genome assemblies.

Using Quast to compare our assembled contigs to the Wilmington reference, we identify two regions with potential assembly errors. These are around positions 185,255 bp to 321,747 bp and 841,954 bp to 907,053 bp, where there is a discontinuity in the alignment of the assembled contigs to the reference genome (S2 Fig), likely caused by difficulty in resolving the genome sequence around these regions during the genome assembly process.

### Pan-genome

We constructed a pangenome from all of the strains described in this paper using Panaroo (Table 4), showing 861 core genes and 6 accessory genes across the 28 strains. 861–865 coding sequences were annotated across the strains, suggesting the majority of genes in *R. typhi* are present in the core genome. Six accessory gene groups were present in only a subset of strains, and we investigated in detail as these represent the only potential non-core genes in the *R. typhi* genome (S1 Text). By aligning sequences from these gene groups to the genome assemblies and identifying the region where genes were missing from assemblies using synteny, we determined that these six groups did not represent real accessory genes which are differentially present in *R. typhi*, but were artifacts of genome assembly (S3–S9 Figs).

### Resequencing of TM2540

TM2540 was previously sequenced and assembled using a combination of Oxford Nanopore MinION and Illumina MiSeq sequencing, with the Oxford Nanopore long reads used to produce a draft assembly which was polished using Illumina data [12]. The work by Kato *et. al.* suggested that this isolate had an unusually high number of variants compared to other strains, therefore we resequenced this isolate using Illumina technology to identify whether the original assembly had spurious SNP calls. We compared the variant calls generated from Snippy using the previous assembly and the newest Illumina MiSeq data and found that the number of SNPs identified using our newer Illumina dataset only is the same as previously described, with 17 SNPs in comparison to other strains. These 17 SNPs are not identified as errors by polishing the genome with Pilon using the newest Illumina data, and TM2540 is positioned with the other modern Laos isolates in our phylogeny. However, when we compare indel calls from Snippy using the new Illumina data and the original assembly, we called three indels using the new Illumina data compared to 20 in the old assembly. After polishing the original assembly with the new Illumina data, we removed 12 of the 20 indels, indicating potential excess indel calls when using the previously published Oxford Nanopore data for assembly.

## Discussion

We demonstrate that the mutation rate across *R. typhi* is extremely low, consistent with previous work [12,13], and the pangenome is almost non-existent with very little accessory genome present across the strains tested. This is consistent with the biology of *R. typhi*, an obligate intracellular bacterium with a reduced genome that typically experiences limited exposure to mutagenic environmental factors and horizontal gene transfer [6]. Recent work has confirmed that lower pangenome fluidity is correlated with the obligate intracellular lifestyle, and is likely due to lifestyle factors and not effective population size, which is also smaller in intracellular parasites [35]. *Rickettsia* genomes show substantial gene loss, common among intracellular organisms that rely heavily on their host for nutrition and other metabolic processes [36]. The lack of genome fluidity in *R. typhi* suggests all inessential genes have been lost from the genome, and that either gene gain via horizontal transfer from other species occurs rarely in *R. typhi,* or selective pressure leads to loss of any inessential genes gained.

Despite the low number of mutations seen across this species, we identified some temporal clustering in the strains examined, with two broad clades representing the historic and modern strains. As all the modern strains in this study were collected from patients in Laos, it is difficult to determine whether the same temporal clustering would be observed with a more geographically diverse data set of modern strains. Notably, there was a single Laos strain which clustered with the historic strains which was most closely related to two historic strains collected in Thailand. It is not possible to determine whether these historic clade strains were continually circulating in Laos, or represents a re-introduction of these strains, potentially from Thailand as closely related strains were demonstrated to be circulating in Thailand in the past. The historical strains used in this study will have undergone many passages in culture, while the modern strains from Laos are low-passage strains sampled as close to possible to the date when the patient presented with *R. typhi* disease. The clustering of a modern Lao strain with the historical strains suggests that the variants seen are circulating in patient strains, and do not represent variation which has arisen through adaptation to culture.

Similarly, our modern Lao strains all have a human origin, but among the historical strains there is no clustering by species of origin, suggesting that *R. typhi* strains do not show distinct genotypes adapted to different mammalian hosts. A weakness of this study is that there are no available genomes from strains obtained directly from fleas, the main vector of *R. typhi*, and it is possible that the genetic diversity of strains in the vector is greater than those which can infect a mammalian host.

We detect one isolate, collected in 1939 in South Africa, with a mutation in the *rpoB* gene, which has previously been implicated in laboratory-induced rifampicin resistance. The mutation we found has not been previously reported to cause rifampicin resistance, and it is unknown whether this mutation contributes to phenotypic resistance in this strain, which was collected before rifampicin was in use. A study comparing the sequences of naturally rifampin-resistance *Rickettsia* to sensitive species hypothesized that specific pairs of residues introducing larger side chain residues could result in reduced binding efficacy for rifampin and lead to resistance [10]. The mutation in the GEAR isolate introduces a larger side chain residue which could lead to steric hindrance of the rifampin binding site. However, the mutations at positions 409 and 973, which are present in all naturally resistant *Rickettsia*, were not seen in any of our isolates.

In this study we used Illumina short-read sequence data to assemble the new strains. Despite the short read length, it was possible to assemble each genome in 11 or fewer contigs, and with only two regions showing possible misassemblies, demonstrating that while long-read sequencing is beneficial for genome assembly it is possible to obtain high-quality genomes with short-read sequencing alone. A target capture sequencing approach using probes designed to hybridise to reference genomes works effectively for *O. tsutsugamushi* [37] and *R. prowazekii* [38]*,* and given the small genome size and limited diversity of *R. typhi* a similar approach could be used to sequence genomes from clinical specimens and potentially from vector hosts. As part of this study, we re-sequenced the TM2540 strain, which has an existing complete genome generated using a hybrid assembly of older Oxford Nanopore and Illumina data. The SNP calls generated from the resequenced strain matched the original assembly, but some of the indels previously identified using Oxford Nanopore data could not be validated using the newer data, suggesting they were artifacts remaining from the assembly process. The previous assembly was generated using R9.4.1 flow cells, which is known to give data with a higher error rate than the newer and more accurate R10 flow cells and kits [39].

The extremely low genetic diversity of *R. typhi* across isolates collected almost a century apart from different continents has implications for investigation into *R. typhi* pathogenesis, diagnostics, treatment, and prevention. Severity in *R. typhi* disease is unlikely to have a pathogen genetic component, and diagnostics which rely on nucleic acid amplification are unlikely to be affected by sequence variation. While rifampicin resistance has been seen in a laboratory strain of *R. typhi* [8]*,* naturally-occurring resistance will need to overcome the low mutation rate and strong selective pressure and, to our knowledge, has not yet arisen in Laos PDR. The lack of variation may also be beneficial in development of *R. typhi* vaccines and point-of-care diagnostic tools, as there is minimal variation in possible antigenic targets.

## Supporting information

S1 Fig A quantitative assessment of the completeness in terms of expected gene content of a genome assembly by BUSCO, showing that the number of complete, fragmented, and missing genes is the same in the complete reference assemblies and our novel assemblies. (TIFF)

S2 Fig Contig alignment results to Wilmington strain by Quast and the Icarus contig viewer. The x-axis represents genome location in the Wilmington genome. Green and blue blocks represent correctly assembled contigs. Red blocks represent misassembled contigs which do not correspond to a single alignment block.
(TIFF)

S3 Fig The illustration of group_506 and group_505 in the reference genomes (Wilmington, B991CWPP, and TH1527) and two selected short-read assembly genomes (AZ306 and AZ331). Positions give the location of this gene in the contig or genome.
(TIFF)

S4 Fig Multiple sequence alignment of group_505 and group_506 sequences. (TIFF)

S5 Fig Alignment of gene group group_506 showing group_506 is absent in short read assembly genomes, AZ306 and TM2522. (TIFF)

S6 Fig The illustration of group_392 and group_524 in assembled genome (NC_006142.1) and short-read assembly genomes (AZ306 and AZ331). (TIFF)

S7 Fig The illustration of group_509 in assembly genome (NC_006142.1) and short-read assembly genomes (TM2607 and TM10184). (TIFF)

S8 Fig The illustration of group_507 and group_525 of assembly genome (NC_006142.1) and short-read assembly genomes (PAKNA, TA837, and TM4234). (TIFF)

S9 Fig Multiple sequence alignment of group_507 and group_525. (TIFF)

S1 Text Supplementary material which references the supplementary figures. (DOCX)
