# Supplementary figures and images for: Complete genomes of Rickettsia typhi reveal a clonal population
Source: PLoS Negl Trop Dis. 2025 Dec 29;19(12):e0013828. doi: 10.1371/journal.pntd.0013828 (PMC12788622; doi:10.1371/journal.pntd.0013828)

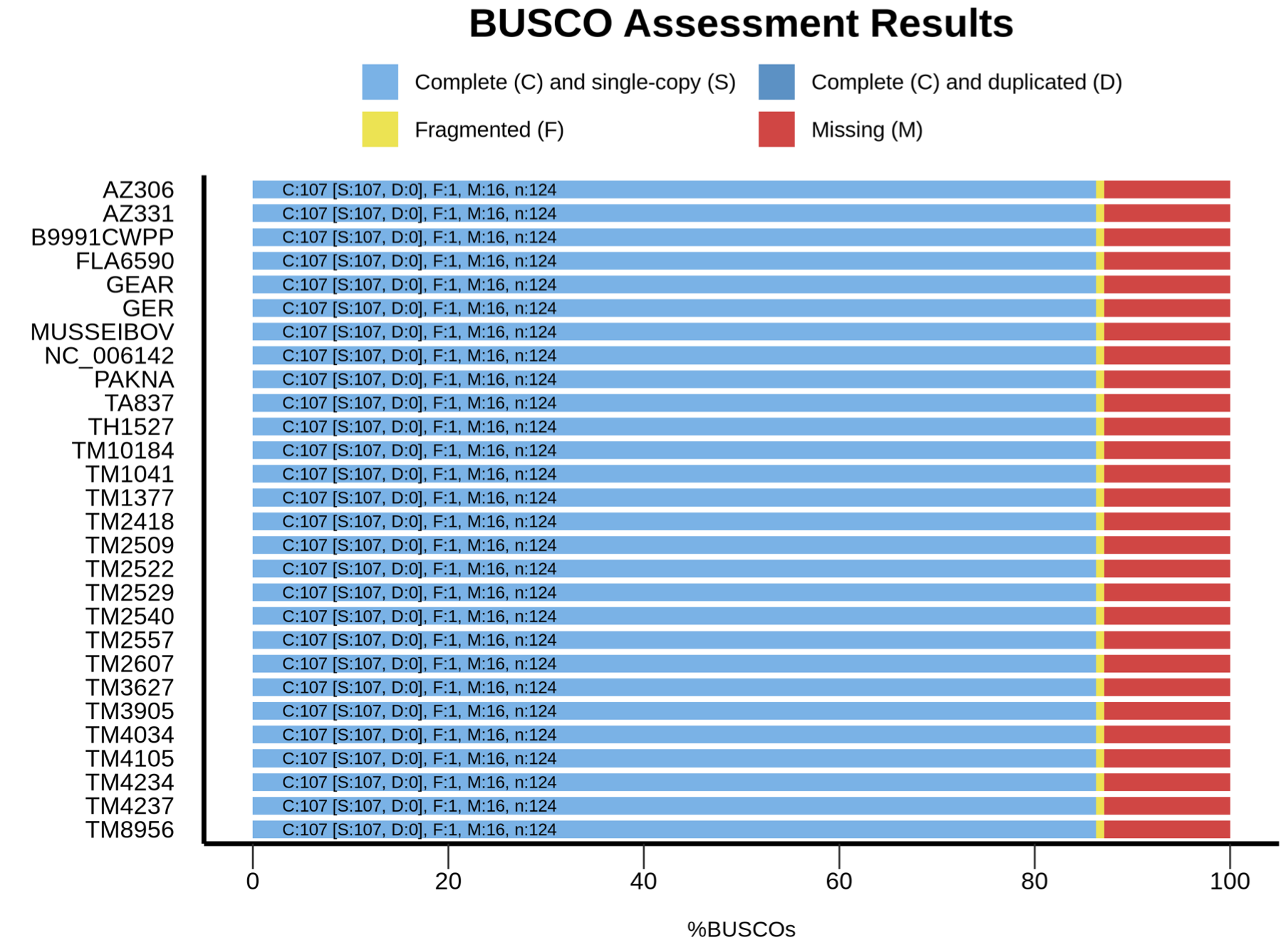

Supplement: S1 Fig — (TIFF) [file pntd.0013828.s001.tiff]

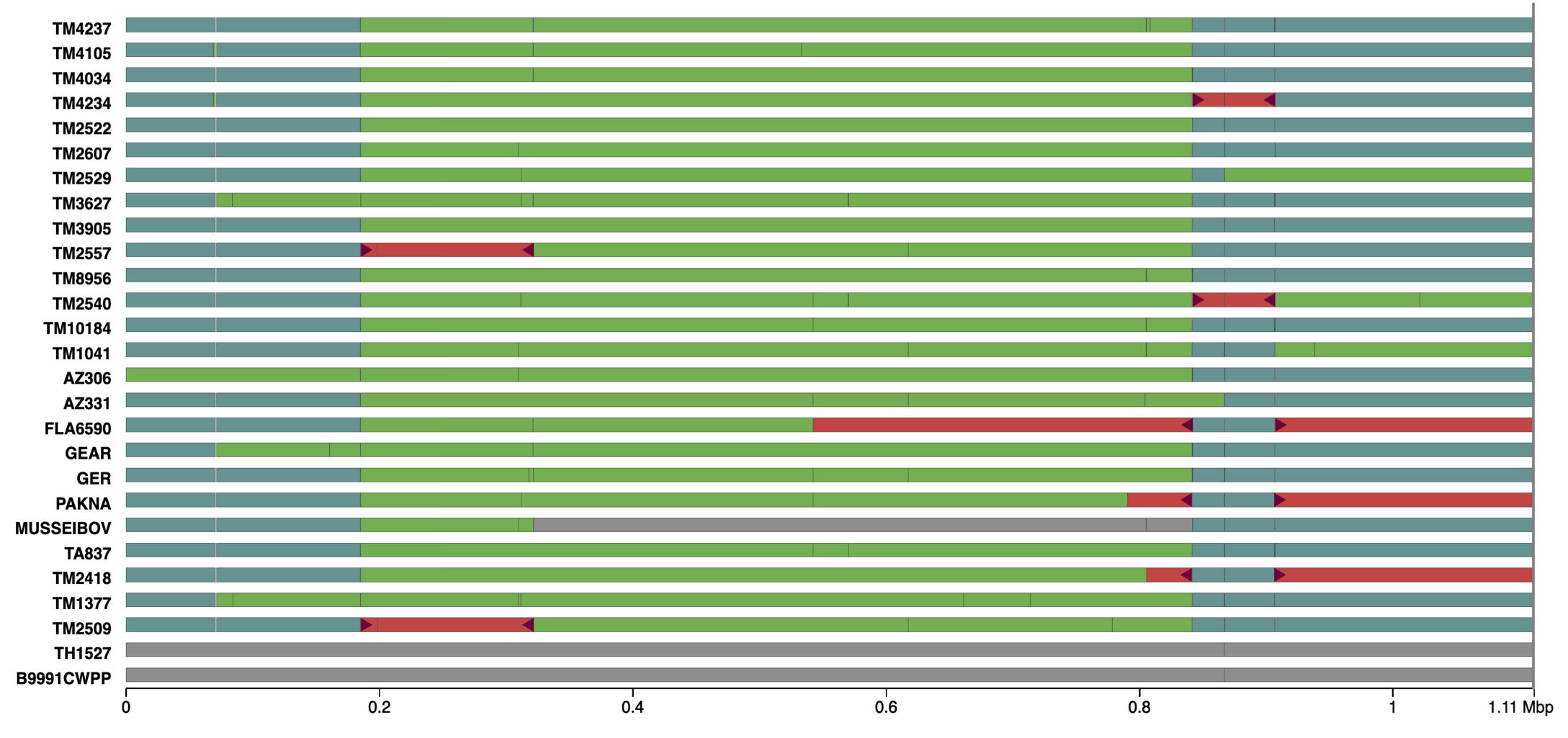

Supplement: S2 Fig — The x-axis represents genome location in the Wilmington genome. Green and blue blocks represent correctly assembled contigs. Red blocks represent misassembled contigs which do not correspond to a single alignment block. (TIFF) [file pntd.0013828.s002.tiff]

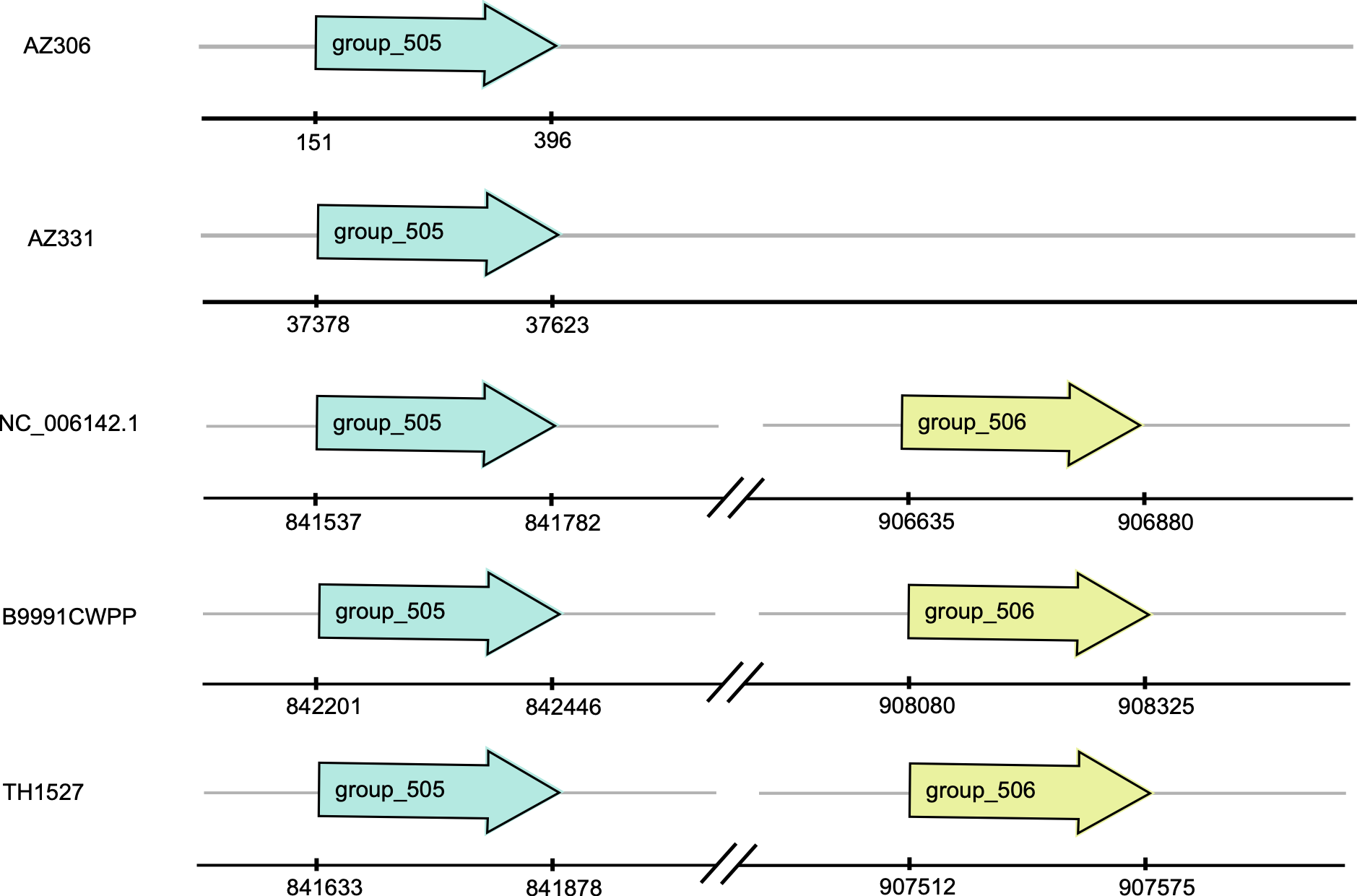

Supplement: S3 Fig — Positions give the location of this gene in the contig or genome. (TIFF) [file pntd.0013828.s003.tiff]

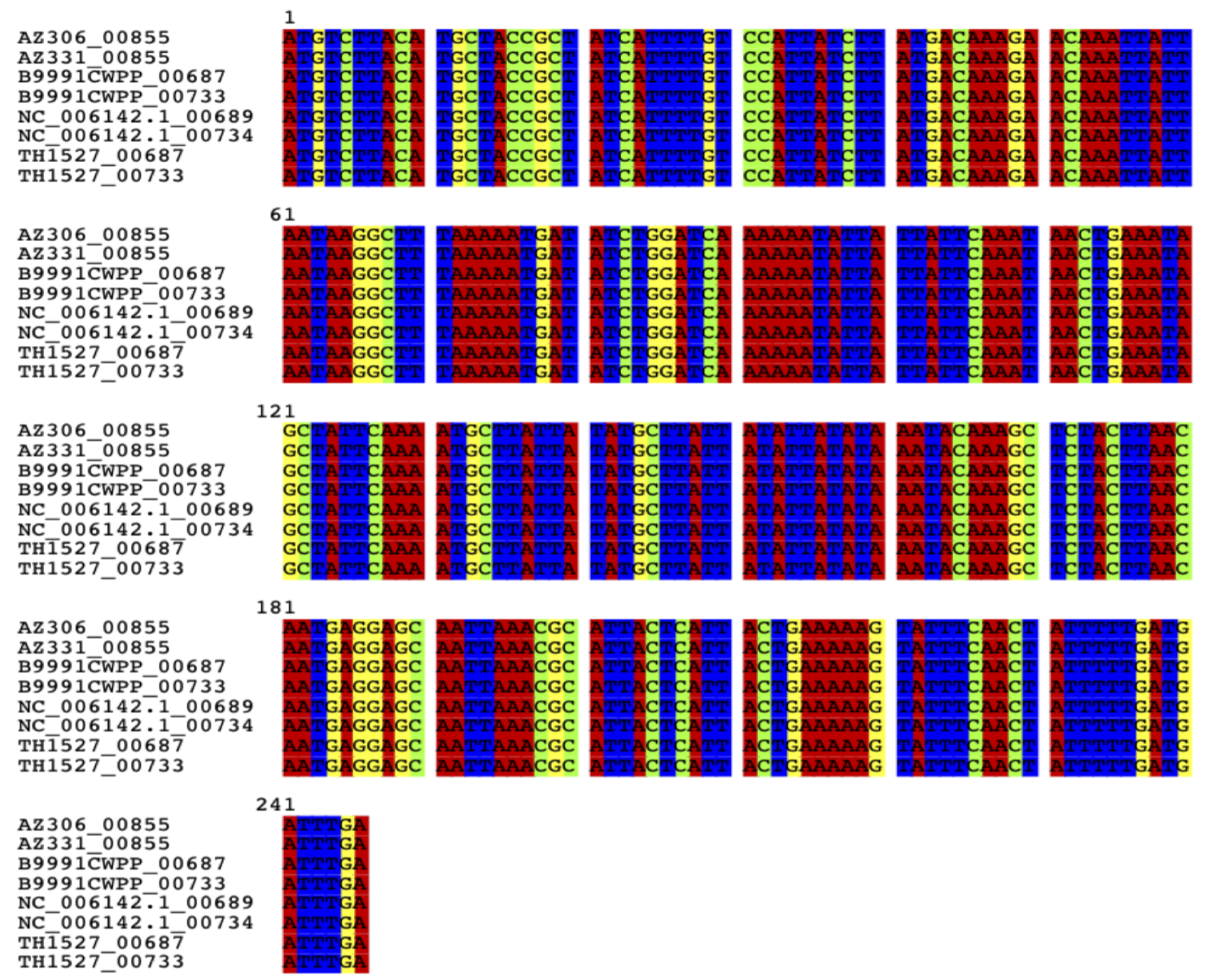

Supplement: S4 Fig — (TIFF) [file pntd.0013828.s004.tiff]

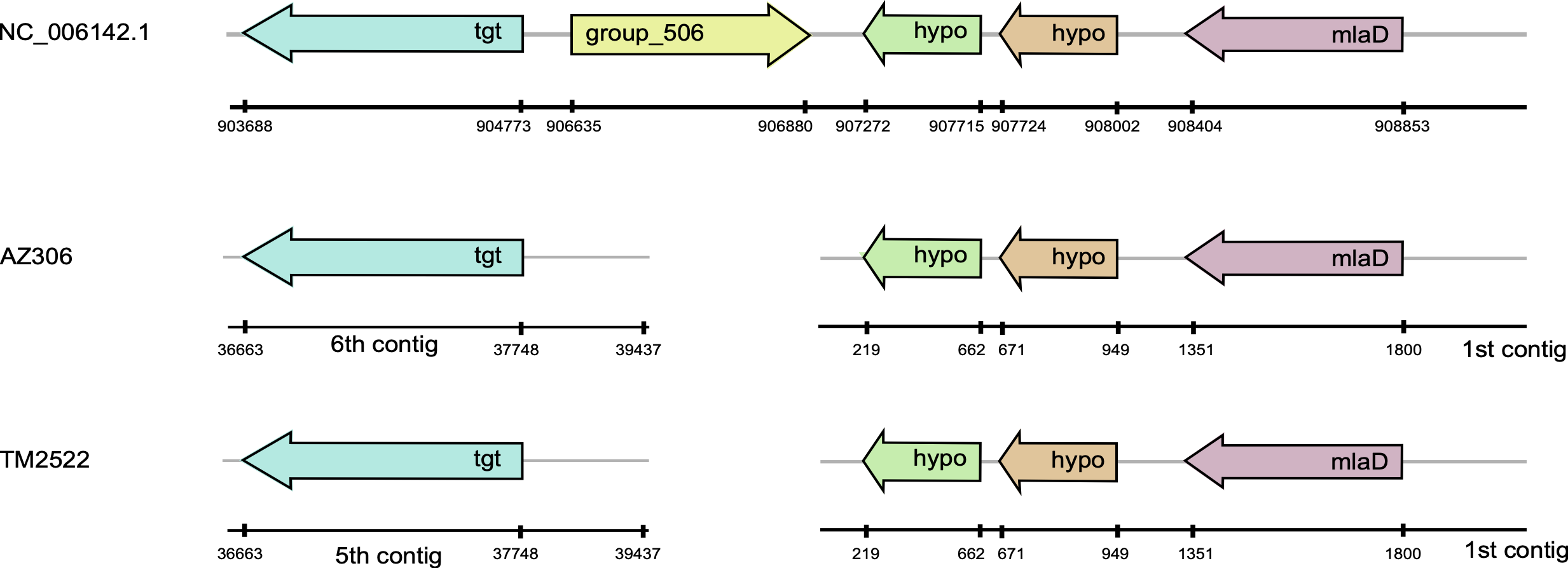

Supplement: S5 Fig — (TIFF) [file pntd.0013828.s005.tiff]

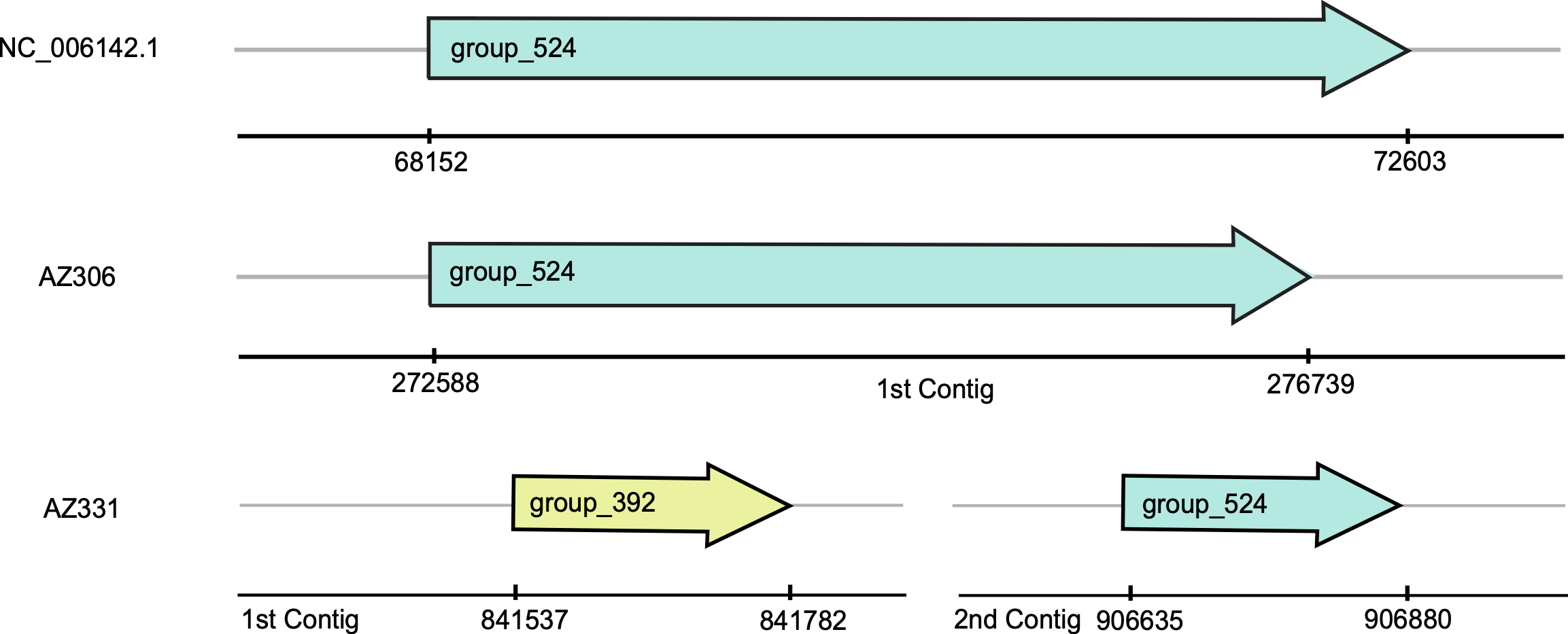

Supplement: S6 Fig — (TIFF) [file pntd.0013828.s006.tiff]

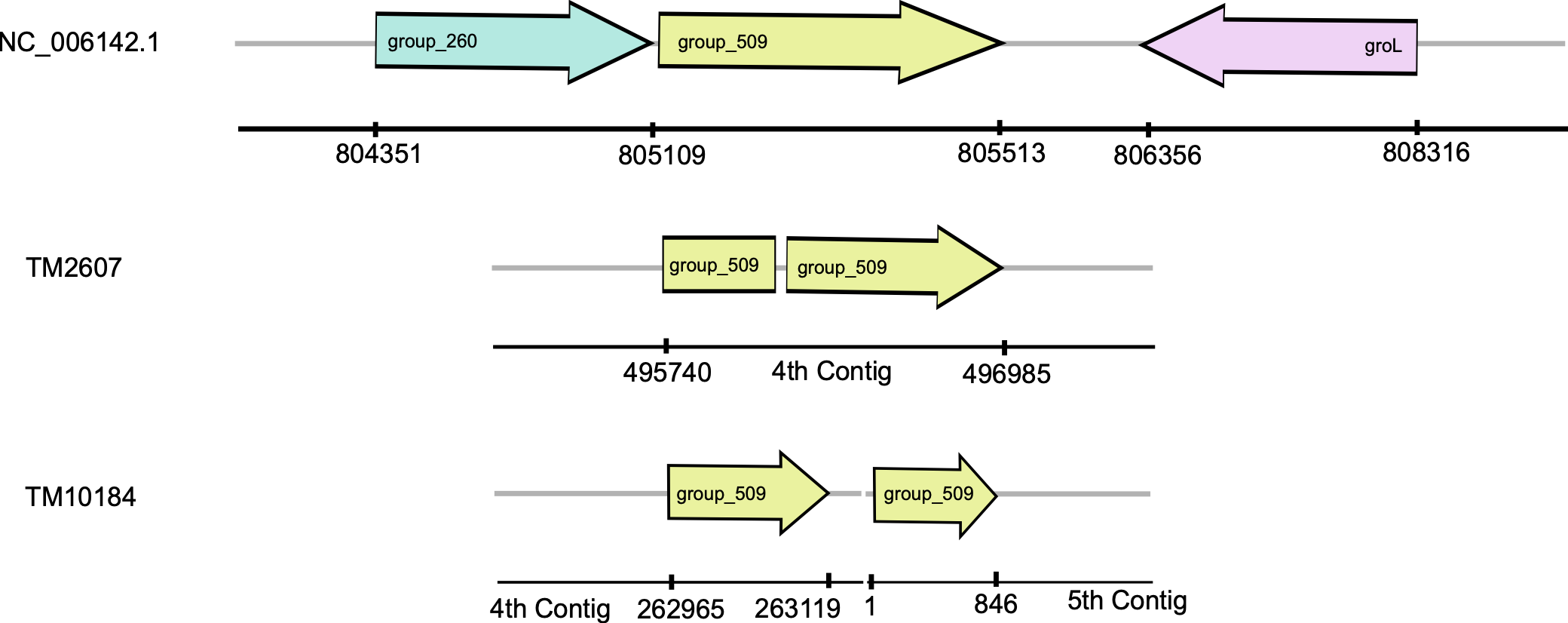

Supplement: S7 Fig — (TIFF) [file pntd.0013828.s007.tiff]

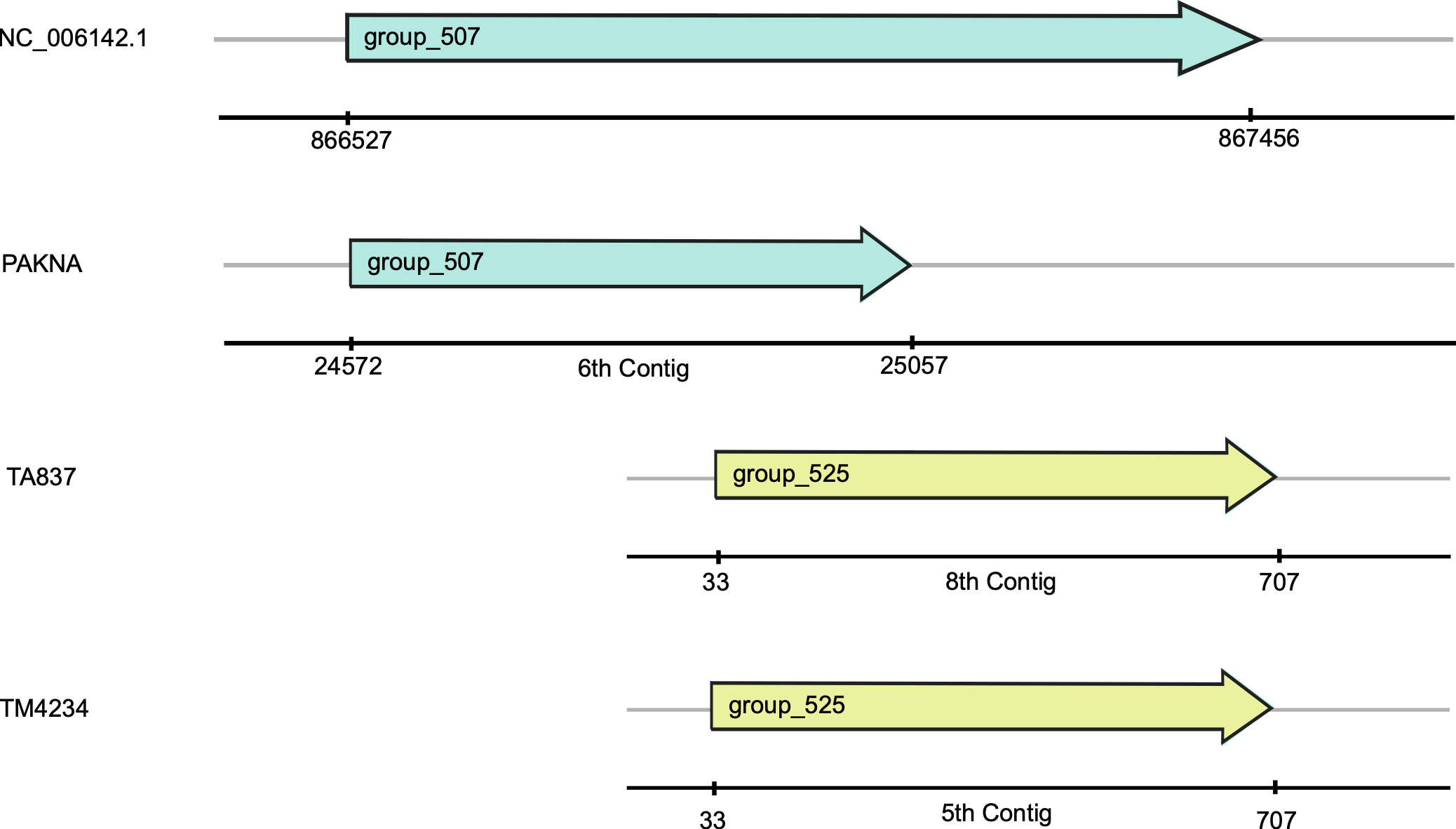

Supplement: S8 Fig — (TIFF) [file pntd.0013828.s008.tiff]

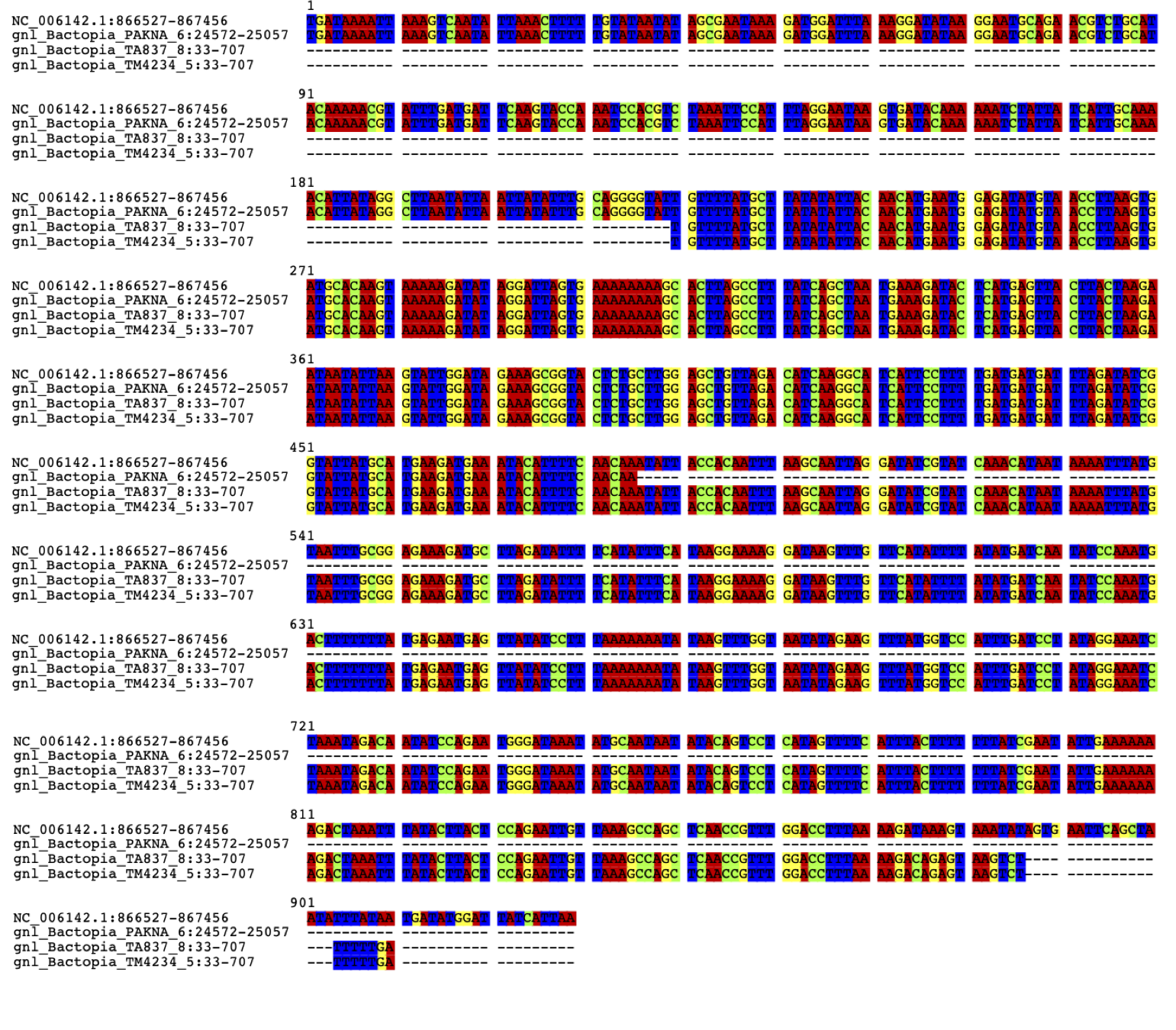

Supplement: S9 Fig — (TIFF) [file pntd.0013828.s009.tiff]
