# Supplementary material for: Complete genomes of Rickettsia typhi reveal a clonal population
Source: PLoS Negl Trop Dis. 2025 Dec 29;19(12):e0013828. doi: 10.1371/journal.pntd.0013828 (PMC12788622; doi:10.1371/journal.pntd.0013828)
Supplement: S1 Text — (DOCX) [file pntd.0013828.s010.docx]

**Supplementary text**

Six gene groups were identified using Panaroo as potential accessory genes, present in less than 100% of the genomes. We investigated each gene group to determine if the gene was really not present in some strains or was missed due to errors in assembly or annotation of the genome, and concluded the genes were missed due to assembly and annotation errors..

1. *Group_506*

Group_506 is a hypothetical protein that is only present in the reference genomes and not the newly assembled strains (S3 Fig). When the sequence of the gene from group_506 in genome NC_006142.1, B9991CWPP, and TH1527 were aligned to all genomes, it was present in all the genomes but with a similar sequence to genes annotated as group_505. Group_505 and group_506 are identical in length, 246bp, and have identical sequences (S4 Fig). Group_506 is likely not present in the Illumina assembly data due to the difficulty of assembling very short duplicated regions using short-read sequencing technology. Using the Wilmington strain as a reference, the flanking genes between group_506 are the *tgt* gene and a hypothetical protein. We found that group_506 is missing from the short read assembly genomes and *tgt* is the last gene located at the end of a contig, indicating that group_506 cannot be assembled (S5 Fig).

1. Group_392

Group_392 is a hypothetical protein that is present in 24 strains, all of which were sequenced by Illumina. The coding sequence of group_392 was present in all the genomes, however in the samples where group_392 was not identified, the sequence was found to form part of the coding sequence of group_524 (S6 Fig). A multiple sequence alignment of group_392 and group_524 shows that group_392 is a shortened fragment of group_524 due to this region being assembled as two separate contigs.

1. Group_509

Group_509 is a bifunctional (p)ppGpp synthetase/guanosine-3'5'-bis(diphosphate) 3’-pyrophosphohydrolase protein. The DNA sequence of the gene from group_509 was present in strain TM2607 but this strain did not have a gene present in group_509 as it is not annotated as having a valid coding sequence. Group_509 lies between group_260 and *groL* genes, and we selected DNA sequences from the end of group_260 to the start of *groL* genes of every absent sample and aligned with group_509 of the reference Wilmington strain. We found that the sequence of group_509 is there, but the contigs are fragmented in strains TM10184 and TM1041, and there is a sequence insertion between two parts of gene in the strains MUSSEIBOV, TM2607, and TM3627 (S7 Fig).

1. Group_507, group_525, and group_512

Group_507 and group_525 are LicD family proteins, and group_512 is a phosphoryl choline transferase LicD protein. Group_507 and group_525 are never found in same strain. In strains where group_525 were found, it is always at the start of the contig, and the comparison of group_507 and group_525 by multiple sequence alignment has shown that group_525 is a shorter, truncated version of group_507; therefore, it is not clustered with group_507. TM2540 assembly data by Illumina lacked group_507, whereas in the TM2540 assembly data by Oxford Nanopore Technology (ONT) group_507 was present (S8 Fig). From the MSA of group_507 and group_512 based on the assembled genomes, the length of both genes is nearly equal, around 800 base pairs, the sequences are very similar, and they are next to each other in the genome (S9 Fig). The missing gene is likely due to the difficulties of assembling this tandem repeat gene.
